# Validation of a Swedish version of the short UPPS-P impulsive behavior scale among young adults

**DOI:** 10.1016/j.abrep.2017.10.001

**Published:** 2017-10-18

**Authors:** Benjamin Claréus, Daiva Daukantaitė, Margit Wångby-Lundh, Lars-Gunnar Lundh

**Affiliations:** Department of Psychology, Lund University, Sweden

**Keywords:** Impulsivity, UPPS, UPPS-P, Swedish, Impulsive behavior scale

## Abstract

The UPPS-P model of impulsivity proposes that impulsivity comprises five distinct facets—negative urgency, positive urgency, lack of premeditation, lack of perseverance, and sensation seeking. The UPPS-P Impulsive Behavior Scale has been used to measure these facets. The purpose of the current study was to develop and evaluate the psychometric properties of a Swedish version of the 20-item UPPS-P Impulsive Behavior Scale (SUPPS-P). The sample comprised 343 Swedish young adults (*M*_age_ = 24.21, *SD* = 2.01; 27% men, 2% other or undisclosed gender identity) who answered a questionnaire including the SUPPS-P; Depression, Anxiety, and Stress Scale (DASS-21); and questions regarding their alcohol consumption and substance use. Confirmatory factor analysis supported a 5-factor, inter-correlated model, where each subscale of the SUPPS-P constitutes one latent variable. The convergent validity was established by replicating previously found correlations between the different impulsivity facets and depression, anxiety, frequency of alcohol consumption, and substance use. The internal consistency was acceptable for all the SUPPS-P subscales (Cronbach's α = 0.65–0.78, McDonald's ω = 0.65–0.79), except lack of perseverance (Cronbach's α = 0.60, McDonald's ω = 0.61). Thus, while the Swedish version of the SUPPS-P is suitable for assessing impulsivity in Swedish young adult samples, further research is needed to improve the psychometric properties of the lack of perseverance subscale.

## Highlights

• A 5-factor inter-correlated model was supported for the Swedish SUPPS-P.
• SUPPS-P subscale correlations to risky behaviors supported the convergent validity.
• Subscale correlations to psychological distress supported the convergent validity.
• All SUPPS-P subscales except lack of perseverance had adequate internal consistency.

## 1 Introduction

In recent years, there has been a growing consensus that impulsivity is a multifaceted construct encompassing various different cognitive and behavioral control mechanisms (Gay, Rochat, Billieux, d'Acremont, & Van der Linden, 2008). Whiteside and Lynam (2001) and Cyders and Smith (2007) developed a model of impulsivity that contains five distinct facets: negative urgency (i.e., acting on impulse when experiencing strong negative affect), positive urgency (i.e., acting on impulse when experiencing strong positive affect), lack of premeditation (i.e., tendency to act without considering the consequences), lack of perseverance (i.e., difficulty in maintaining focus during difficult or boring tasks), and sensation seeking (i.e., pursuing exciting or potentially perilous activities). All of these facets can be measured with the UPPS-P Impulsive Behavior Scale (Lynam, Smith, Whiteside, & Cyders, 2006).

Subsequent research has shown that these five facets of impulsivity are associated with distinct psychopathologies and various risk and addictive behaviors, thus establishing the discriminant validity and utility of the UPPS-P model (Cyders and Smith, 2007, Miller et al., 2003, Whiteside et al., 2005). For example, sensation seeking and lack of premeditation/perseverance predict the frequency of engagement in risky behaviors such as drinking and substance use, whereas urgency is more strongly related with problematic engagement in these behaviors (Coskunpinar et al., 2013, Magid and Colder, 2007, Smith et al., 2007, Verdejo-García et al., 2007). Previous research has further demonstrated that participants scoring high in urgency and a lack of perseverance tend to exhibit elevated levels of depression and anxiety (Billieux et al., 2012, Kämpfe and Mitte, 2009). With regards to gender differences, Cyders (2013) established the gender measurement invariance of the UPPS-P model of impulsivity. However, some gender differences were in fact found, with males scoring significantly higher on sensation seeking and positive urgency and significantly lower on negative urgency compared to females (Cyders, 2013, d'Acremont and Van der Linden, 2005).

Because the original UPPS-P scale is rather lengthy (59 items), researchers have recently been attempting to shorten it. Currently, there are two validated versions of a 20-item UPPS-P. One version was created by selecting four items for each facet that had the highest factor loadings for that facet from the original UPPS-P; it has been validated in French (Billieux et al., 2012), Italian (D'Orta et al., 2015), and Spanish (Cándido, Orduña, Perales, Verdejo-García, & Billieux, 2012). However, this approach to the selection of items was criticized by Cyders, Littlefield, Coffey, and Karyadi (2014), as it reduced the content validity of the original impulsivity model (cf. Smith, McCarthy, & Anderson, 2000). In their version, Cyders et al. (2014) instead aimed to retain satisfactory construct coverage by selecting the items from the long scale that had the highest corrected item-total correlations, and then deleting redundant items (i.e., those with inter-item correlations of > 0.50 with the selected item); this process was then repeated to select the remaining three items for each subscale. They then validated this new 20-item UPPS-P in an American sample, demonstrating that it had sound psychometric properties, which were comparable to the full-length version. This abridged 20-item version by Cyders et al. (2014) saved an estimated 6.50 to 9.75 min compared to the 59-item version, suggesting that it is a promising research tool for lowering participant burden while retaining content validity.

We aimed to evaluate the psychometric properties (factor structure, internal consistency, and convergent validity) of the Swedish version of SUPPS-P, developed by Cyders et al. (2014). The convergent validity was assessed by replicating previously found relationships between the impulsivity facets and risky behaviors such as alcohol and substance use, as well as psychological distress (i.e., depression, anxiety, and stress). We hypothesized that Swedish young adults' lack of perseverance and their negative and positive urgency would correlate with elevated levels of psychological distress, and that their lack of premeditation/perseverance and sensation seeking would correlate with higher frequency of alcohol consumption and be higher in young adults who had ever taken another drug besides alcohol. Additionally, young men are expected to score higher on sensation seeking and positive urgency than are young women, whereas women are expected to score higher on negative urgency.

## 2 Materials and methods

### 2.1 Participants and procedure

The sample comprised 343 participants (239 women, 92 men, and 7 other or undisclosed gender identity) with a mean age of 24.21 years (*SD* = 2.01); 306 participants reported studying, 29 working, 4 job-seeking, and 1 an internship as their primary occupation. We also included questionnaire data from 5 participants without demographic data due to technical problems.

This study was part of a larger project about health and well-being among Swedish young adults. Participants were primarily recruited by publishing advertisements on social media sites, posting on billboards around the Lund University campus, and e-mail dispatches to students currently enrolled at Lund University. Individuals aged 20–27 were invited to partake in an online survey in exchange for one cinema ticket or two lottery tickets. Participants completed a battery of questionnaires including demographic questions, the SUPPS-P, and the 21-item Depression, Anxiety, and Stress Scale (DASS-21). Seven control items (e.g., “Please answer ‘Disagree’ to this question”) were included throughout the full-length survey to screen for inattentive and negligent responding. Because previous research has demonstrated that removing inattentive participants improves reliability and power (Maniaci & Rogge, 2014), four participants who failed to provide correct responses to two or more of these control questions were consequently excluded from the data analysis. The study was approved by Lund University's ethical review board (no. 2016/1059).

### 2.2 Measures

Impulsivity was measured via the Swedish version of the SUPPS-P (available upon request from the corresponding author). Currently, a validated Swedish version of the UPPS Impulsive Behavior Scale containing only four subscales (negative urgency, lack of premeditation, lack of perseverance, and sensation seeking) is available (Marklund, 2008). Therefore, the Swedish translations of the items included in Cyders et al.'s version of these subscales were selected from this scale for use in the current study. Because the positive urgency subscale was not included in this Swedish version of the UPPS scale, these four items were translated from English to Swedish, whereupon a bilingual Swedish-English speaker back-translated the items into English and resolved any discrepancies in translation. We also administered the DASS-21 (Lovibond & Lovibond, 1995), along with items enquiring how often the participant drank alcohol (1 = *Never*, 2 = *Once a month or less frequent*, 3 = *2–4 times a month*, 4 = *2 or 3 times a week*, 5 = *> 4 times a week*) and whether they had ever taken drugs other than alcohol (*Yes* or *No*).

### 2.3 Statistical analyses

To determine the factor structure of the Swedish SUPPS-P scale, we tested three models using confirmatory factor analysis (CFA) with maximum likelihood estimation and robust standard errors. The analyses were performed with the lavaan package (version 0.5–23.1097) of R (Rosseel, 2011). Model A specified a 5-factor model with inter-related latent variables corresponding to each SUPPS-P subscale. Model B added a higher-order latent “impulsivity” trait to the first model. Finally, Model C was based on previous research where positive and negative urgency were distinct factors loading onto a higher “emotion-based rash action” trait; lack of premeditation and perseverance were distinct factors loading onto a higher-order “deficits in conscientiousness” trait; and sensation seeking constituted an independent impulsivity factor (Billieux et al., 2012, Cyders et al., 2014). The residuals of items 6 and 8 were covaried in all models because these items were phrased similarly.

Goodness of fit was evaluated using the χ^2^ statistic, where a nonsignificant value represents an acceptable fit. However, as the χ^2^ statistic can inflate type II error for large samples (Brown, 2014), we also computed several approximate fit indices with conventional cutoffs: the comparative fit index (CFI), root mean square error of approximation (RMSEA), and standardized root mean square residual (SRMR). Acceptable fit standards are generally CFI ≥ 0.90, RMSEA ≤ 0.08, and SRMR ≤ 0.10 (Hu & Bentler, 1999). As Models B and C are nested within Model A, a χ^2^ difference test was used to evaluate which model had the better fit.

Internal consistency was calculated using Cronbach's α and McDonald's ω, with the latter being more robust than the former if the assumption for tau-equivalence is not met (Dunn, Baguley, & Brunsden, 2014). Pearson point-biserial correlations were used to assess the effects of ever having taken a drug other than alcohol (*no* = 0, *yes* = 1) and gender (*women* = 0, *men* = 1) on the SUPPS-P and DASS-21 subscales. Because of the small subgroup size (*n* = 5), participants whose gender was other or undisclosed were excluded from these analyses. Furthermore, two-tailed Pearson's correlations were used to assess relations between the impulsivity facets and depression, anxiety, stress, and frequency of alcohol consumption.

## 3 Results

**Fig. 1:**
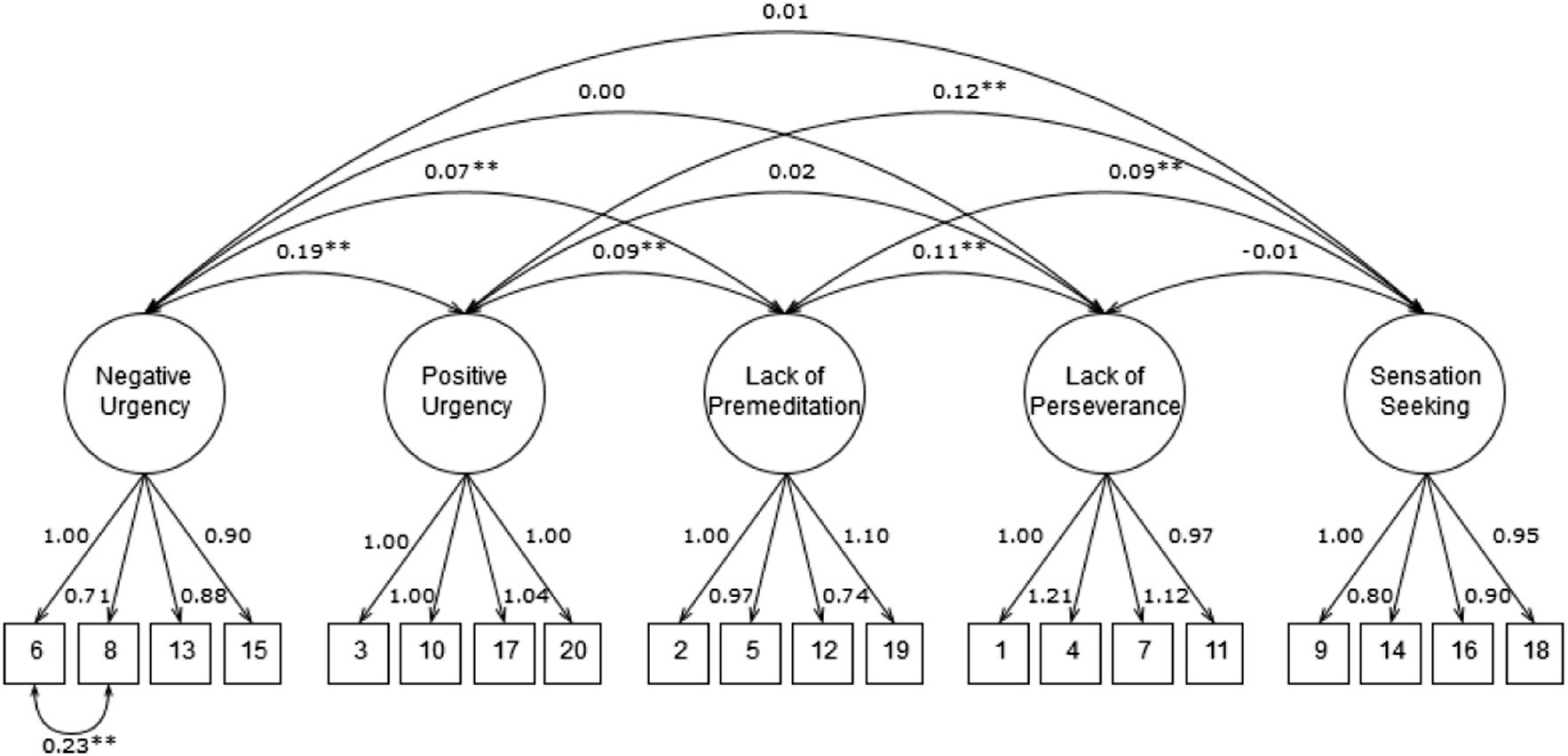
Factor loadings and covariances of the five-factor inter-correlated model of impulsivity, measured by the SUPPS-P. *Note.* **p < 0.01.

The CFA revealed that the five-factor inter-correlated model (Model A; Fig. 1) had an acceptable fit, χ^2^(159) = 343.69, CFI = 0.88, RMSEA = 0.06, SRMR = 0.07. In comparison, Model B [χ^2^(164) = 422.37, CFI = 0.82, RMSEA = 0.07, SRMR = 0.09] and Model C [χ^2^(164) = 384.40, CFI = 0.85, RMSEA = 0.07, SRMR = 0.08] had a slightly worse fit. The χ^2^ difference testing confirmed that Model A had a significantly better fit than did Model B [χ^2^(5) = 82.82, *p* < 0.001] and Model C [χ^2^(5) = 46.97, *p* < 0.001].

Means, standard deviations, internal consistencies, and correlation coefficients are presented in Table 1. All scales used in this study had acceptable or borderline acceptable Cronbach's α and McDonald's ω coefficients, except for the lack of perseverance subscale (α = 0.60, ω = 0.61). Significant gender differences were found for sensation seeking and negative urgency: men scored higher than did women on sensation seeking, and women scored higher than did men on negative urgency. Additionally, participants who had ever taken a drug besides alcohol scored higher on all impulsivity facets except for negative urgency when compared to those who had not. Frequency of alcohol consumption was significantly positively correlated with lack of premeditation, lack of perseverance, and sensation seeking.

**Table 1:** Descriptive statistics, internal consistencies, and Pearson's correlations between the SUPPS-P impulsivity facets and other variables.

Variable		M (SD)	Range	α	ω	1.	2.	3.	4.	5.	6.	7.	Gender (*N* = 331)	DrugTest (N = 338)	FreqAlcohol (*N* = 338)
1.	SUPPS-P: negative urgency	1.09 (0.62)	0.00–3.00	0.65	0.65								− 0.11*	0.07	0.02
2.	SUPPS-P: positive urgency	0.57 (0.58)	0.00–3.00	0.78	0.78	0.49**							0.05	0.19**	0.08
3.	SUPPS-P: lack of premeditation	0.85 (0.56)	0.00–2.50	0.78	0.79	0.14**	0.30**						0.00	0.24**	0.30**
4.	SUPPS-P: lack of perseverance	0.89 (0.48)	0.00–2.50	0.60	0.61	− 0.04	0.04	0.43**					0.02	0.17**	0.11*
5.	SUPPS-P: sensation seeking	1.40 (0.72)	0.00–3.00	0.69	0.67	− 0.03	0.26**	0.17**	− 0.04				0.23**	0.13*	0.19**
6.	DASS-21: depression	4.62 (4.60)	0–20	0.89	0.89	0.45**	0.26**	0.06	0.05	− 0.12*			− 0.03	0.02	− 0.12*
7.	DASS-21: anxiety	3.57 (3.23)	0–18	0.71	0.73	0.36**	0.33**	0.01	− 0.03	− 0.05	0.56**		− 0.02	0.03	− 0.02
8.	DASS-21: stress	7.12 (4.30)	0–21	0.82	0.81	0.50**	0.27**	0.02	− 0.09	− 0.18**	0.56**	0.56**	− 0.14*	− 0.03	− 0.07

Note. N = 343 unless otherwise specified. Dummy variables in Pearson's point-biserial correlations were coded as women = 0 and men = 1 for gender and never tested = 0 and had tested = 1 for whether the participant had ever tested another drug besides alcohol (DrugTest).

SUPPS-P = Short UPPS-P Impulsive Behavior Scale, DASS-21 = short Depression, Anxiety, and Stress Scale, FreqAlcohol = Frequency of alcohol consumption (ranging from 1 = *Never* to 5 = *> 4 times a week*). α = Cronbach's alpha, ω = McDonald's omega.

* *p* < 0.05, ** p < 0.01.

## 4 Discussion

The present study examined the psychometric properties of a Swedish version of the SUPPS-P in a sample of young adults. The CFA confirmed that a model consisting of five inter-correlated latent variables—negative urgency, positive urgency, lack of premeditation, lack of perseverance, and sensation seeking—had the best fit. These results are consistent with previous research using an identical scale by Cyders et al. (2014) as well as with the different language versions of the other SUPPS-P (Billieux et al., 2012, Cándido et al., 2012, D'Orta et al., 2015). Furthermore, moderate correlations were found between negative and positive urgency, and between lack of premeditation and lack of perseverance, upholding past findings that these constructs have a latent relationship (Billieux et al., 2012, Cyders et al., 2014).

Several unique correlations were found in the present study. As in Billieux et al.’s study (2012), negative urgency was significantly correlated with depression and anxiety in this study; however, positive urgency was significantly correlated with psychological distress in this study, but not in Billieux et al.'s. We also replicated results by Coskunpinar et al. (2013) and Magid and Colder (2007) by showing that individuals high in lack of premeditation, lack of perseverance, and sensation seeking had a higher frequency of alcohol consumption. Although we did not distinguish between one-time use, frequent use, or abuse of different substances, we nevertheless found that lack of premeditation and positive urgency were higher in individuals who had ever taken another drug besides alcohol. These results are in line with previous studies demonstrating that a lack of premeditation is a significant predictor of substance use (Lynam & Miller, 2004), and that positive urgency can predict illicit drug use (Zapolski, Cyders, & Smith, 2009). Additionally, men scored significantly higher on sensation seeking than did women, and the direction and effect size of this bivariate relationship converges with previous research findings (Cyders, 2013, d'Acremont and Van der Linden, 2005). However, the results did not support Cyders et al.'s (2013) findings that men tend to show higher positive urgency than do women; but the results do accord with those of d'Acremont and Van der Linden (2005), who showed that women scored higher than did men in negative urgency. The results of our findings have limited generalizability, however, as the current sample was not balanced in terms of gender.

Other study limitations include that the negative urgency, lack of perseverance, and sensation seeking subscales had lower internal consistencies than has previously been reported in research with any version of the SUPPS-P (Billieux et al., 2012, Cándido et al., 2012, Cyders et al., 2014, D'Orta et al., 2015). This is especially true for the lack of perseverance subscale. The low internal reliability in this scale might explain why the current study failed to replicate positive correlations between lack of perseverance and anxiety/depression (Billieux et al., 2012, Kämpfe and Mitte, 2009). This result could be explained by the fact that, while the current sample was comparable in age to Cyders et al.’s American, Billieux et al.’s French, and Cándido et al.'s Spanish sample, it did not exclusively comprise students. Further, as Cyders et al. (2014) selected items for their version of the SUPPS-P based on item content in order to provide satisfactory coverage of the construct, the lowered reliability could be a consequence of reducing the number of observations while not systematically omitting items with the highest error variances (Smith et al., 2000). Additional studies should focus on validating the SUPPS-P in Swedish populations older than 27 years, especially considering that D'Orta et al. (2015) found significant negative correlations between age and all five SUPPS-P subscales, and in samples with an even gender distribution.

In conclusion, the current study supported measurement invariance and sound psychometric properties of the SUPPS-P scale in Swedish young adults, showing promise as a useful tool for assessing impulsivity in research settings, although more research is needed regarding the lack of perseverance subscale.

## Role of funding sources

Funding for this study was provided by Swedish Research Council for Health, Working Life and Social Welfare (FORTE; Dnr 2016-00248). FORTE had no role in the study design, collection, analysis or interpretation of the data, writing the manuscript, or the decision to submit the paper for publication.

## Contributors

DD, LGL, and MWL designed the larger project within which this study was conducted. BC conducted the literature search and provided summaries of previous research studies. BC, in collaboration with DD, conducted the statistical analyses. BC wrote the first full draft of the manuscript. BC, DD, LGL, and MWL have all edited several subsequent drafts of the paper. All authors contributed to and have approved the final manuscript.

## Conflict of interest

We declare that we have no conflicts of interest in the authorship or publication of this contribution.
